# A case of phyllodes tumor with rapid growth during pregnancy and lactation period: a case report

**DOI:** 10.1186/s40792-024-01895-w

**Published:** 2024-04-24

**Authors:** Shiori Tohyama, Yoshiya Horimoto, Yumiko Ushiyama, Ryoko Semba, Shiori Hotchi, Naomi Sugano, Kanako Ogura, Fumi Murakami

**Affiliations:** 1 Department of General Surgery, Juntendo University Nerima Hospital, Tokyo, Japan; 2 Department of Breast Oncology, Faculty of Medicine, Juntendo University, 2-1-1 Hongo, Bunkyo-Ku, Tokyo, 1113-0033 Japan; 3 Department of Breast Oncology and Surgery, Tokyo Medical University, Tokyo, Japan; 4 Department of Diagnostic Pathology, Juntendo University Nerima Hospital, Tokyo, Japan; 5 Department of Radiology, Juntendo University Nerima Hospital, Tokyo, Japan

**Keywords:** Phyllodes tumor, Pregnancy, Lactation

## Abstract

**Background:**

The age of onset of the phyllodes tumor is generally in the late 40 s, and diagnosis and treatment during pregnancy and lactation are rare. We herein present a case of a phyllodes tumor that rapidly increased in size during the pregnancy and lactation period.

**Case presentation:**

A 39-year-old woman was referred to our hospital with a mass in the right breast that increased in size during the pregnancy and lactation period. On ultrasound (5 week postpartum), a well-defined lobulated mass with internal septations and fluid retention was observed. Magnetic resonance imaging of the breast at 8 week postpartum revealed a 70-mm-sized smooth-margin mass with multilocular cystic components. Marked proliferation of stromal cells with high cell density was observed in a biopsy specimen taken at the previous hospital. We diagnosed the mass as a phyllodes tumor of borderline malignancy and excised it at 13 week postpartum. The excised tumor was 85 mm in diameter and its interior was filled with a milk-like substance. Histologically, there was only a mild increase in stromal cell density but fibrosis with associated degeneration was prominent. The final diagnosis was benign phyllodes tumor with degeneration.

**Conclusions:**

We report a case of a phyllodes tumor that rapidly increased in size during pregnancy and the lactation period. The accumulation of a milk-like substance was thought to be responsible for the rapid growth of the tumor.

## Background

Phyllodes tumor is classified by the WHO as a type of fibroepithelial tumor, and can be categorized based on histological features into benign, borderline, and malignant [1]. Regardless of its nature, surgical excision is the standard treatment [2]. The age of onset is typically in the late 40 s [2] and diagnosis and treatment during pregnancy are rare. In this report, we present a case of a phyllodes tumor that rapidly increased in size during the pregnancy and lactation period.

## Case presentation

**Fig. 1:**
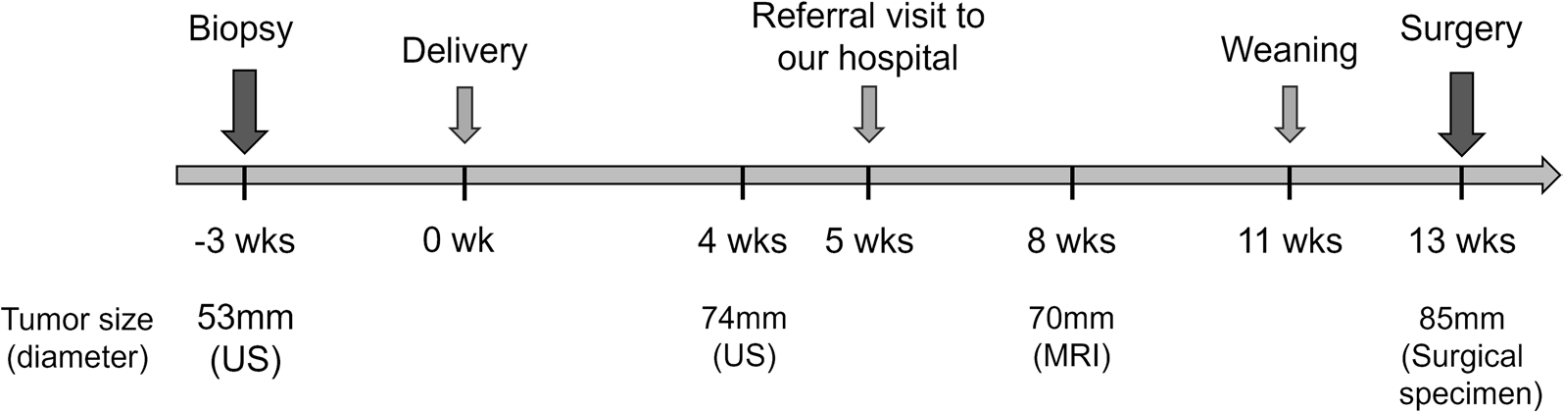
Clinical course and changes in tumor size. Clinical course and changes in tumor size are shown. Wk; week, US; ultrasound, MRI; magnetic resonance imaging

A 37-year-old woman was found with a right breast mass by a screening breast ultrasound. The solid oval-shaped mass was diagnosed as a fibroadenoma and the patient was followed up at the previous hospital. Two years later, when the woman attended a check-up at 10 months of pregnancy, the mass increased to a diameter of 50 mm. A needle biopsy was performed leading to a histological diagnosis of fibroepithelial lesion. Due to her pregnancy, the tumor excision was planned after delivery. Following childbirth, during lactation, the tumor grew further, and at 4 week postpartum, she expressed a desire for tumor excision and was referred to our hospital. She had a history of hyperthyroidism, managed through observation only, and there was no relevant family history. An overview of the clinical course is shown in Fig. 1.

**Fig. 2:**
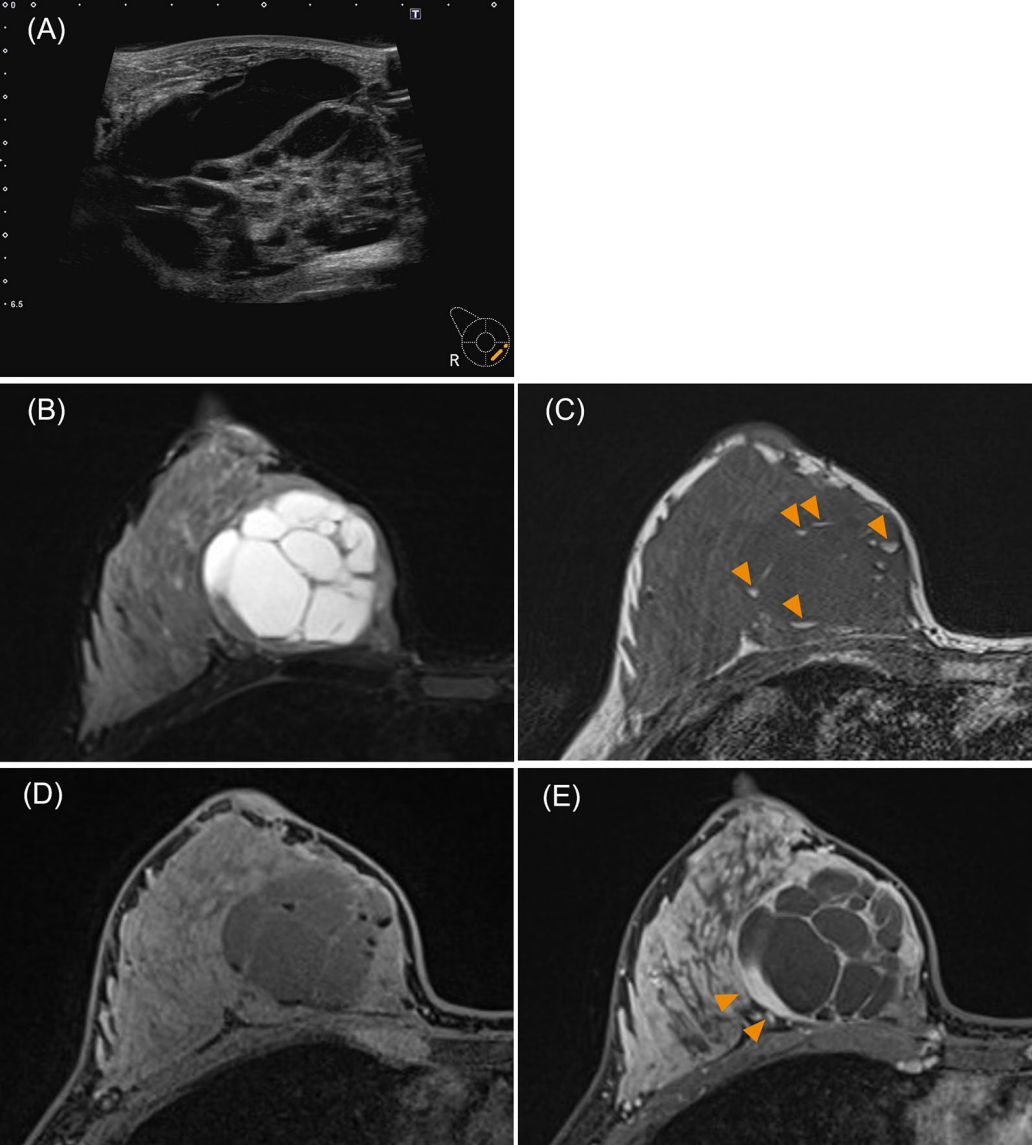
Findings of pre-surgical imaging. **A** Findings of ultrasound at the initial visit to our hospital (5 week postpartum). A well-defined lobulated mass with clear borders is observed in the right lower inner quadrant. Internal septations are present, and there is evidence of fluid retention. **B**–**E** Findings of the breast MRI performed 2 weeks before surgery (11 week postpartum). The tumor is exerting pressure outward, displacing the breast tissue. **B** T2-weighted image with fat suppression. The T1-weighted image (**C**) showed a high-signal area in the fluid component within the cyst (arrow), and this signal was suppressed in the fat-suppressed image (**D**). **E** Enhanced T1-weighted image with fat suppression in the late phase after contrast. An arrow indicates a solid part along the edge of the tumor

**Fig. 3:**
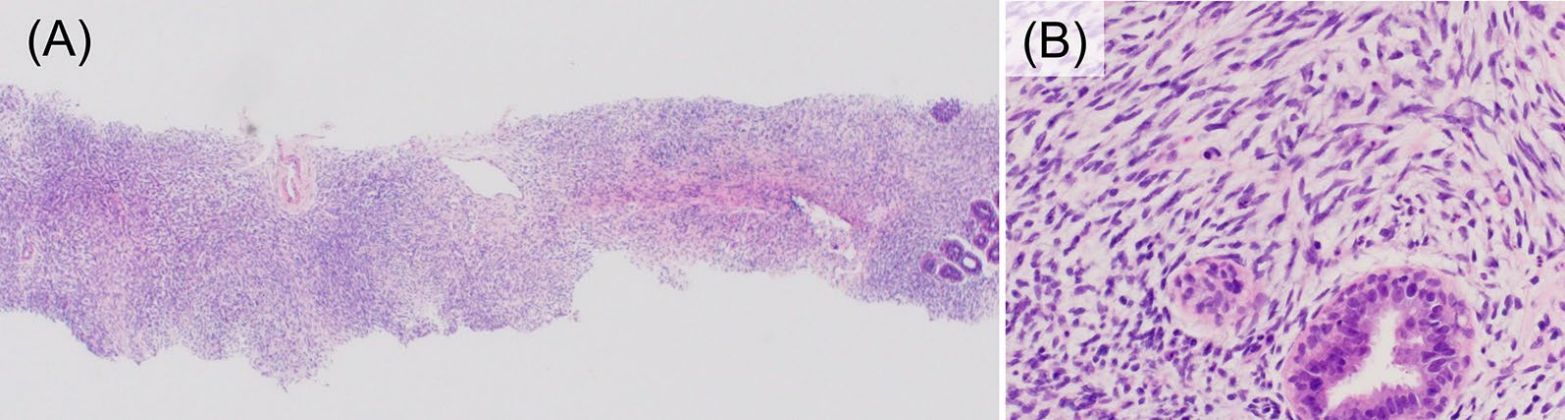
Histological findings of needle biopsy. Histological findings of needle biopsy at low (**A**) and high (**B**) magnification (× 40 and × 400, respectively). Marked proliferation of stromal components with high cell density was observed but the cellular atypia is mild, and there are few mitotic figures

Upon the initial visit to our hospital (5 week postpartum), an ultrasound revealed a well-defined lobulated mass with internal septations and fluid retention and the longest diameter was not measurable (Fig. 2A). A breast magnetic resonance imaging (MRI) at 8 week postpartum showed a 70-mm-sized smooth-margin mass with multilocular cystic components (Fig. 2B–E). Although hematoma, abscess and metastatic tumor are among the differential diagnoses for polycystic masses, the former two were discarded because signs suggestive of an accumulation of blood or pus were not found in the fluid area. Moreover, a metastatic tumor was also discounted as each septum was thin and had a smooth surface. The liquid component of the cystic structure contained a high-signal and fat-suppressed area on T1-weighted images, indicating a floating fat component (Fig. 2C, D), consistent with milk-like substance. There was a small solid part along the edge of the tumor. This area showed a fast-and-persistent contrast pattern (Fig. 2E) and no diffusion loss (diffusion coefficient: 1.23), suggesting that this part was predominantly composed of stromal components. The histological findings of the biopsy conducted at the previous hospital are presented in Fig. 3. Marked proliferation of stromal cells with high cell density was observed. Thus, we diagnosed the mass as a phyllodes tumor of borderline malignancy, although the initial diagnosis at the previous hospital was a fibroepithelial lesion. Due to the histological diagnosis and the tumor's tendency to grow, it was excised 13 week postpartum.

**Fig. 4:**
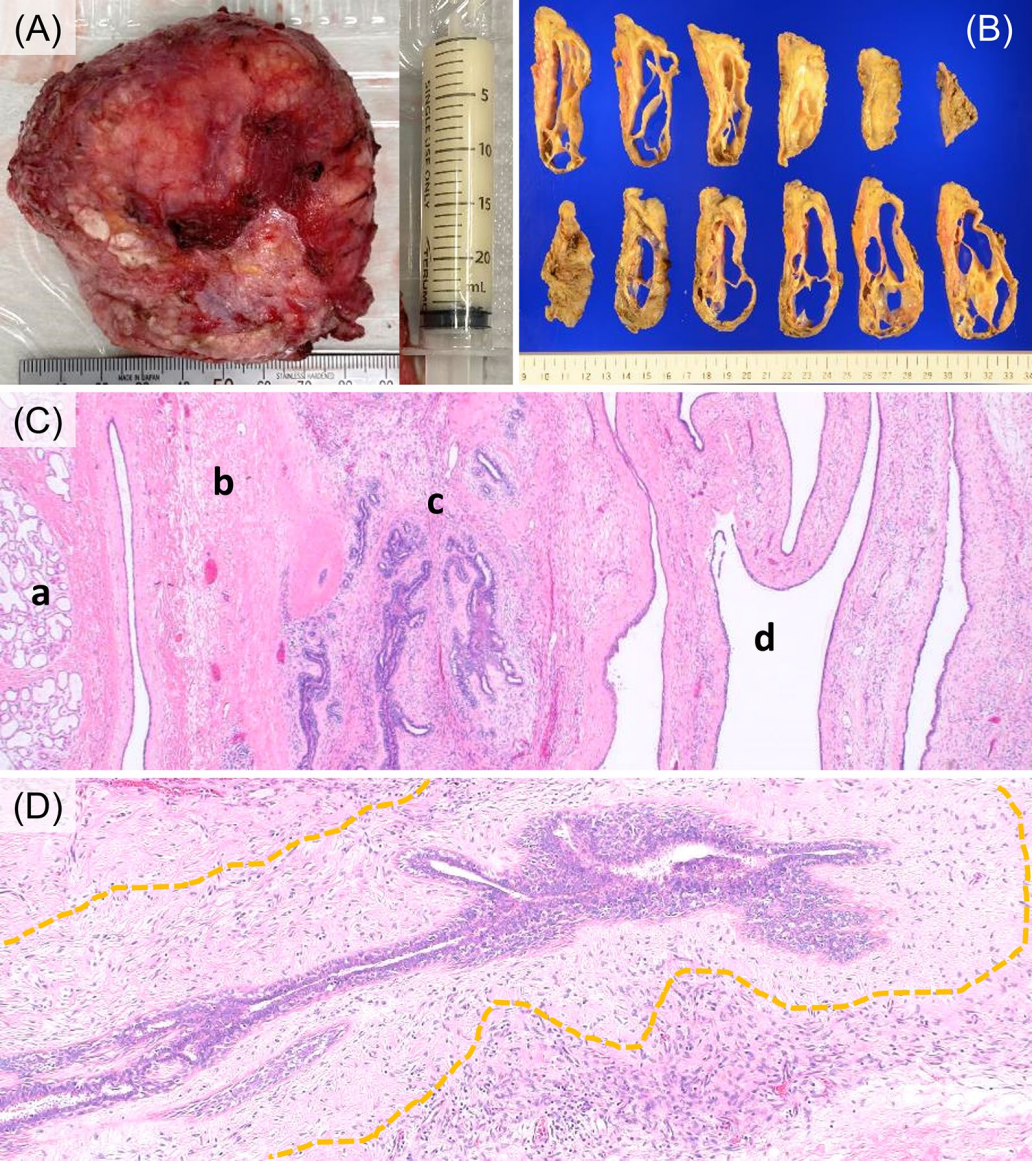
Histological findings of surgical specimen. **A** Excised specimen and aspirated fluid. **B** Cut surface of the formalin-fixed surgical specimen. **C**, **D** Histological findings from hematoxylin–eosin-stained specimens. **C** a; Normal lactating mammary tissue, b; sparse area with edematous changes near the normal mammary gland, c; slightly dense area with degeneration, d; cystic structure. **D** Only few cells around the ducts (circled by the yellow dotted line) due to degenerative change

The excised tumor was 85 mm in diameter and its interior was filled with fluid. An aspiration of the fluid revealed a milk-like substance (Fig. 4A). The size of the excised specimen was 110 × 80 × 32 mm and the cut surface revealed a thin-walled, multilocular cystic tumor (Fig. 4B). Histologically, there was only a mild increase in stromal cell density but degenerative and fibrotic changes were more prominent in the stromal component of the tumor, probably due to oppression (Fig. 4C, D). A phyllodes tumor is generally characterized by a particularly high cell density of stromal cells around the ducts, whereas in the present tumor, there were comparatively few stromal cells around the ducts due to the degenerative change (Fig. 4D). Taken together, the final diagnosis was benign phyllodes tumor with degeneration.

## Discussion

In this case, we describe a phyllodes tumor that grew from the pregnancy period through childbirth to the lactation period. A systemic review assessed 43 cases of PT during pregnancy and lactation [3]. According to the publication, 79.5% of examined cases were characterized by a rapid growth before surgery. Notably, there was no relationship between rapid growth and malignancy, suggesting that its growth may be related to the hormonal milieu during gestation and/or lactation, rather than an indicator of malignancy [3]. The review included 26 cases (60.5%) of malignant PT. Malignant phyllodes tumors during pregnancy have also been reported numerously by other authors [4, 5] However, whether phyllodes tumors diagnosed during pregnancy are truly more malignant or not remains unclear, taking into account the publication bias. To draw a conclusion, a larger epidemiological investigation based on healthcare statistics data, including comparisons with non-pregnant women, is necessary.

The aforementioned review also discussed ultrasound findings, such as tumor shape, but there was no mention of intra-tumoral findings. In a recent case report that conducted serial ultrasound measurements of a benign phyllodes tumor during pregnancy and lactation, the largest growth was observed during early pregnancy and no obvious milk retention was noted upon removal. [6]. Meanwhile, Likhitmaskul et al. reported the case of a 36-year-old woman who was diagnosed with a 20 cm-sized benign phyllodes tumor at 32 week gestation, whose internal cystic cavity contained about 300 mL of milky liquid when the surgical specimen was resected [7]. Likewise, in our case, the surgical specimen had accumulated a milk-like liquid and presurgical MRI images revealed prominent fluid retention. Hence, the rapid enlargement during the lactation period was considered to be mainly due to the accumulation of breastmilk produced by the mammary duct epithelium inside the tumor.

In the current case, the biopsy specimen showed a marked proliferation of stromal components with high cell density. However, in the surgical specimen, the increase in stromal cell density was just mild, and fibrosis with associated degeneration was more prominent. We suspect that the observed difference in findings may be attributed to the rapid filling of the cystic cavity with milk-like components, leading to partial ischemia and detachment of stromal cells, resulting in fibrosis.

Reports of enlargement of a phyllodes tumor during pregnancy are prevalent, yet thorough considerations on the causes are lacking. Due to alterations in hormonal milieu such as serum estrogen, breasts undergo substantial physiologic changes during pregnancy including vascular hyperplasia and proliferation of alveoli and lobules [3, 8–10]. However, no evidence has been produced that a phyllodes tumor may be influenced by steroid hormones. A previous study suggested that stromal estrogen receptor (ER)-α promoted tumor growth via promoting angiogenesis [11] but stromal cells in mammary tissue are generally negative for ER by immunohistochemistry. We also confirmed that stromal cells in all phyllodes tumors and fibroadenoma examined were negative for ER in a recent study [12]. Moreover, because of the inherent tendency of phyllodes tumors to grow rapidly, a rigorous comparison with tumors in non-pregnant women is necessary to determine whether growth is accelerated during pregnancy.

## Conclusions

We report a case of a phyllodes tumor that rapidly enlarged during pregnancy and the lactation period. Because of rapid tumor growth and the biopsy finding of borderline malignancy, the patient underwent surgical resection as soon as possible after delivery. Milk retention should be considered as one of the reasons for a rapid increase in phyllodes tumors during pregnancy in daily practice.

## Data Availability

Not applicable.

## Abbreviations

ER: Estrogen receptor
MRI: Magnetic resonance imaging

## References

[1] Tumours B (2019). WHO classification of tumours.

[2] Mishra SP, Tiwary SK, Mishra M, Khanna AK (2013). Phyllodes tumor of breast: a review article. ISRN Surg.

[3] Alipour S, Eskandari A, Johar FM, Furuya S (2020). Phyllodes tumor of the breast during pregnancy and lactation: a systematic review. Arch Iran Med.

[4] Zhang WX, Kong XY, Zhai J, Fang Y, Song Y, Wang J (2021). Fatal outcome of malignant phyllodes tumor of the breast in pregnancy: a case and literature review. Gland Surg.

[5] Faulds TT, Bruckner J, Mousa M, Bhanu S, Chin M, Cendrowski K (2024). Giant phyllodes tumor of the breast: a case report. Radiol Case Rep.

[6] Ooi LY, Lim GH, Gudi MA (2022). Effect of pregnancy and lactation on a benign phyllodes tumour. BMJ Case Rep.

[7] Likhitmaskul T, Asanprakit W, Charoenthammaraksa S, Lohsiriwat V, Supaporn S, Vassanasiri W, Sattaporn S (2015). Giant benign phyllodes tumor with lactating changes in pregnancy: a case report. Gland Surg.

[8] Nejc D, Pasz-Walczak G, Piekarski J, Pluta P, Bilski A, Sek P, Potemski P, Durczynski A, Wronski K, Jeziorski A (2008). Astonishingly rapid growth of malignant cystosarcoma phyllodes tumor in a pregnant woman: a case report. Int J Gynecol Cancer.

[9] Yu JH, Kim MJ, Cho H, Liu HJ, Han SJ, Ahn TG (2013). Breast diseases during pregnancy and lactation. Obstet Gynecol Sci.

[10] Lee SS, Hartman HJ, Kuzmiak CM, Crosby KL (2013). The management of breast symptoms in the pregnant and lactating patient. Curr Obstet Gynecol Rep.

[11] Péqueux C, Raymond-Letron I, Blacher S, Boudou F, Adlanmerini M, Fouque MJ, Rochaix P, Noël A, Foidart JM, Krust A, Chambon P, Brouchet L, Arnal JF, Lenfant F (2012). Stromal estrogen receptor-α promotes tumor growth by normalizing an increased angiogenesis. Cancer Res.

[12] Yuan M, Saeki H, Horimoto Y, Ishizuka Y, Onagi H, Saito M, Hayashi T, Arakawa A, Yao T (2023). Stromal Ki67 expression might be a useful marker for distinguishing fibroadenoma from benign phyllodes tumor of the breast. Int J Surg Pathol.

